# Practitioners’ best practices to Adopt, Use or Abandon Model-based Testing with Graphical models for Software-intensive Systems

**DOI:** 10.1007/s10664-022-10145-2

**Published:** 2022-05-30

**Authors:** Emil Alégroth, Kristian Karl, Helena Rosshagen, Tomas Helmfridsson, Nils Olsson

**Affiliations:** SERL Sweden Blekinge Institute of Technology, Karlskrona, Sweden

**Keywords:** Model-based testing, Test automation, Software Engineering, Industrial study, Expert knowledge

## Abstract

Model-based testing (MBT) has been extensively researched for software-intensive systems but, despite the academic interest, adoption of the technique in industry has been sparse. This phenomenon has been observed by our industrial partners for MBT with graphical models. They perceive one cause to be a lack of evidence-based MBT guidelines that, in addition to technical guidelines, also take non-technical aspects into account. This hypothesis is supported by a lack of such guidelines in the literature.

**Objective:** The objective of this study is to elicit, and synthesize, MBT experts’ best practices for MBT with graphical models. The results aim to give guidance to practitioners and aspire to give researchers new insights to inspire future research.

**Method:** An interview survey is conducted using deep, semi-structured, interviews with an international sample of 17 MBT experts, in different roles, from software industry. Interview results are synthesised through semantic equivalence analysis and verified by MBT experts from industrial practice.

**Results:** 13 synthesised conclusions are drawn from which 23 best-practice guidelines are derived for the adoption, use and abandonment of the technique. In addition, observations and expert insights are discussed that help explain the lack of wide-spread adoption of MBT with graphical models in industrial practice.

**Conclusions:** Several technical aspects of MBT are covered by the results as well as conclusions that cover process- and organizational factors. These factors relate to the mindset, knowledge, organization, mandate and resources that enable the technique to be used effectively within an organization. The guidelines presented in this work complement existing knowledge and, as a primary objective, provide guidance for industrial practitioners to better succeed with MBT with graphical models.

## Introduction

Model-based testing (MBT) based on graphical models is a testing technique where models are driven by MBT test code to generate and, if used for automated testing, run test cases (Utting et al. 2012). Graphical models are in this context models of edges and vertexes that define test behavior whilst MBT test code is specialized code used to run the models. Furthermore, a test case is defined as a sequence of actions that aim to verify how well a system under test (SUT) conforms to its requirements. The technique is often defined as a black-box technique and has several inherent benefits which stem from models defining tests on a higher logical and/or semantic level of abstraction than, for instance, test code. This abstraction reduces technical and cognitive complexity of the tests—tests are easier to overview and understand (Khan et al. 2019). These models vary in type and appearance depending on domain, context or purpose, i.e. they can be textual (e.g. Gherkin (North 2010)), formal (e.g. mathematical) (Broy et al. 2005) or graphical models (e.g. graphs) (Utting et al. 2012; Kramer and Legeard 2016).

This study delimits its scope to automated testing with graphical models—henceforth when discussing MBT, if not stated otherwise, it primarily refers to MBT with graphical models—where system states and state-transitions are visualized using graphical objects (i.e. vertexes), lines and/or arrows (i.e. edges). These models, for example defined in UML (Lima and Faria 2015), are drawn in modelling tools, e.g. YeD (Aho et al. 2011), or within the MBT tools themselves, e.g. Graphwalker (Zafar et al. 2021). The study’s delimitation stems from our industrial partners’ needs to get better understanding of MBT. Additionally, we decided to limit the scope of the study since investigating all types of MBT was considered too resource intensive for the study.

In practice, after a model has been developed, it is exported or developed into an executable format, i.e. MBT test code components. These components consists of code that implement the behavior of the modelled graphical, commonly referred to as model drivers. When developed manually, such components may be programming language specific, but for certain test drivers/tools (e.g. RT-Tester (Peleska 2013) or Testar (Rueda et al. 2015)) the components may be considered programming language agnostic. The MBT test driver (i.e. code in this discussion) can be written to interact with the SUT on different levels of abstraction, e.g. unit, application programming interface (API) or graphical user interface (GUI) level. The connection between code and graphical, i.e. vertexes or edges, in the model enables reuse of test code by copying graphical objects in the model or by connecting two objects with a new or existing state transition. Additionally, this setup provides the technique with inherent architectural design support for the tests, which helps improve the tests’ maintainability and reusability.

MBT has been researched for over 40 years, resulting in a myriad of solutions, as shown in multiple systematic literature reviews and mapping studies as well as taxonomies on the topic (Iqbal et al. 2018; Gurbuz and Tekinerdogan 2018; Nguyen et al. 2017; Jin and Lano 2021; Utting et al. 2012). A recent tertiary study has also been conducted that classifies these secondary studies (Villalobos-Arias et al. 2019). The tertiary study concludes that MBT, including but not limited to graphical models, is primarily studied from a technical perspective—studies focus on modelling with UML or state-transition models for unit or integration tests. Additionally, the study concludes that there are missing areas within empirical MBT research. This includes more studies into MBT tools (Zafar et al. 2021), to increase its impact and adoption in industrial practice (Schneider 2021). One stated reason for the limited adoption is a lack of industrial success stories and empirical studies (Vásquez et al. 2019; Martınez 2019; Villalobos-Arias et al. 2019). Another reason is the limited focus on evidence-based studies that also consider non-technical MBT aspects—practical and organizational guidelines for how to adopt, use and abandon the technique.

In this study we aim to help bridge this gap in knowledge through an international deep-interview study with 17 experts in MBT with graphical models. The goal of the study is to elicit and synthesize best practice guidelines from the experts’ experiences from adoption, use and abandonment of the technique in industry. Primarily, we seek experiences from successful projects rather than lessons learned from failed projects. To achieve the objective, our study, classified as exploratory but primarily descriptive, is not delimited to technical best practices but also includes non-technical aspects associated with the process and organization for successful MBT.

The result of the study is a set of 23 synthesized best practices for MBT with graphical models. These results are verified by MBT experts who determine them to be generally applicable within the domain of software-intensive systems. In this study, software-intensive systems refers to software systems and applications developed for end users (Freeman and Hart 2004), excluding embedded systems and software built to be used by other systems. We do not exclude that the results are applicable in other domains but lack support to make any such claims.

The main contributions of this paper therefore are:

1. Experience reports on the adoption, use and abandonment of MBT in industrial practice from 17 international experts,
2. A set of 13 generalized conclusions/observations about MBT adoption, use and abandonment in industrial practice, and,
3. 23 verified best practices for how to adopt, use and abandon^1^ MBT in industry.

The remainder of this paper is structured as follows: Section 2 will present related work and an extended motivation for the study. The paper continues in Section 3 with a description of the research methodology. Section 4 presents the results of the study, followed by Section 5 that will present the derived best practices. In Section 6, the results will be discussed and, finally, the paper will be concluded in Section 7.

## Related work and extended motivation for this study

Model-based testing has received a lot of attention from research the last 40 years, as evident from systematic literature reviews, mappings and tertiary studies on the topic. In a tertiary study from 2019 by Villalobos-Arias et al. (Villalobos-Arias et al. 2019), the authors analyzed 22 secondary studies—systematic literature reviews and systematic mappings—to identify current areas of MBT research. They found five research areas; SUT, test objectives, model specification, test generation and test execution. The study also found that MBT has been applied in most domains of software engineering but the studies primarily focus on unit and integration tests. Hence, while technical aspects associated with MBT are covered, organizational, human or other softer factors are underrepresented. We identify this as an important gap in research since these factors are highly relevant for the technique’s adoption, use and longevity in practice. Support for this claim is taken from the tertiary study, which identified that there are many tools available for MBT but still the application of the technique is low in practice (Schneider 2021). This can be explained by the many technical challenges connected to the technique, but also unknown non-technical challenges. These non-technical challenges—associated with softer factors—thereby warrant further empirical research to uncover their existence, extent and how they can be addressed.

Looking closer at the secondary studies, for example Iqbal et al. (Iqbal et al. 2018), who performed a broad literature review of empirical MBT research in 2018, evaluated the quality of MBT research. Although finding many papers, and showing that the research area is mature, their analysis found that only half of the analyzed papers (N = 87) contained the necessary details to properly evaluate their quality. Furthermore, although all papers were empirical, only 19 papers contained industrial data, such as model specifications, and only 3 used industrial code. The remaining 68 papers did either not specify the origin of the used specifications or code, or used academic, open source or artificially constructed examples. The conclusion drawn from the review is thereby that more industry-focused, or co-produced, research is required to judge MBT’s industrial applicability. Hence, another gap in knowledge that this study helps to cover.

Another literature review is presented by Gurbuz and Tekinerdogan (Gurbuz and Tekinerdogan 2018) who focused their study on MBT’s use for testing software qualities, in particular software safety. The review identified 36 industrial examples, but almost half (N = 15) were conducted in either the automotive or railroad domain and none of the studies included human subjects. Hence, further evidence that most MBT studies focus on technology/tools despite the fact that many of the papers state that they aim to study the technique’s use in practice. As such, many studies fail to provide a complete view of the stated studied phenomenon since the human perspective, e.g. user experience, test processes and best practices, are not considered. Further support for the lack of industrial involvement is given by a literature review by Nguyen et al. (Nguyen et al. 2017). In their review, the authors found that 41 out of 48 (i.e. 90 percent) of papers on testing security of cyber-physical systems, are written solely by academic researchers. Whilst this observation does not reflect how well the studies’ are grounded in industrial data, it provides indicative support to the lack of co-production in MBT research.

A more recent literature review by Jin and Lano (Jin and Lano 2021), looked at a twenty year sample of papers (N = 443) published between 1999 and 2019 that focused on model types, intermediate formats between models to tests, and coverage criteria. The outcome of the study are future research directions, deficiencies and trends. Similarly to previously mentioned reviews, the study concludes that technological choices are premiered, in particular modelling schemes.

User-oriented research does however exist. For instance, Utting et al. (Utting et al. 2012) define a user-oriented taxonomy that aims to support decision-making when selecting MBT tools. The taxonomy covers different types of MBT aspects, including model specification, test generation and test execution. The authors’ explicitly state that they exclude aspects concerning ease of use, performance, interoperability, maintainability and traceability, but cover the perspectives of subject selection, redundancy, test characteristics, MBT as a test paradigm, test selection criteria, technology selection and mode of execution (online/offline). The taxonomy is, as stated, tailored towards MBT users, evaluated by mapping existing approaches from research onto the taxonomy. Albeit more encompassing than many previous studies, their study still has a technological focus, neglecting several human aspects, e.g. process and organization, which are perceived important for efficient/effective MBT. The taxonomy has also been extended in 2016 by Felderer et al. (Felderer et al. 2016; Felderer et al. 2011) who added classification of model-based security testing (MBST). This work takes environmental and other dimensions into account, but focuses on a product perspective and its security, omitting human perspectives such as development best practice, processes and communication. These taxonomies were later complemented by the tertiary study by Villalobos-Arias et al. (Villalobos-Arias et al. 2019) and their results on MBT’s application, which provided a general overview of the area.

Due to the maturity of MBT, much knowledge has also been synthesized in books. For example, in their book, “The craft of Model-based testing”, Jorgensen (Jorgensen 2017) presents a comprehensive guide to model-based testing, focusing primarily on how to construct models, different types of models and tools but also cover aspects such as the adoption and use of MBT in practice. Here, the evidence-based results of this study provide confirmatory support.

Despite the vast body of knowledge, many studies on MBT are still referencing a lack of evidence about MBT from an industrial perspective (Janicki et al. 2012), e.g. lack of industrial experience reports and success stories. In a study from 2021 by Garousi et al. (Garousi et al. 2021), an empirical and industrial study is presented where multiple MBT tools are evaluated. Among the contributions of this work are lessons learned and obstacles of adopting and using MBT in practice. A possible explanation to the lack of industrial studies can be attributed to a slow adoption, and interest, from industry (Schneider 2021). Reasons for this slow adoption include the need to adopt new ways-of-working and that MBT tools are incompatible with other tools, environments and common ways-of-working (Li et al. 2016; Dias Neto et al. 2007). Therefore, other techniques, for instance Behavioral-driven development (BDD), which builds on similar foundations as MBT but are more similar to traditional script-based testing, have gotten more traction in practice (Li et al. 2016). Another perceived root-cause to the limited success of MBT is a lack of knowledge about the technique in industry. More knowledge transfer is therefore perceived as necessary (Dias Neto et al. 2007). However, it is important to note that the amount of adoption of the technique varies between domains. For instance, within safety- and reliability-critical domains such as avionics (Peleska et al. 2018; Hemmati et al. 2018), automotive (Drave et al. 2018) and financial technology (Nikiforova et al. 2021; Asaadi et al. 2012), MBT is more common. One explanation for this phenomenon is that these systems often include high degrees of low-level message passing with high combinatorial complexity, which MBT effectively mitigates.

In summary, the body of knowledge on MBT is vast. However, when looking into the literature, most papers concern technical MBT solutions, whilst empirical studies, and industrial experience reports, are more limited (Janicki et al. 2012). Studies on best practices and softer factors associated with the technique are also lacking. In this paper, we address this gap in knowledge by eliciting insights, experiences and observations from expert users of the technique that have used MBT in various industrial settings for one or several years. This provides an academic contribution in terms of showing how MBT operates in industrial practice, but also best-practices for industrial practitioners that seek to adopt, better use, or abandon MBT.

## Methodology

**Fig. 1:**
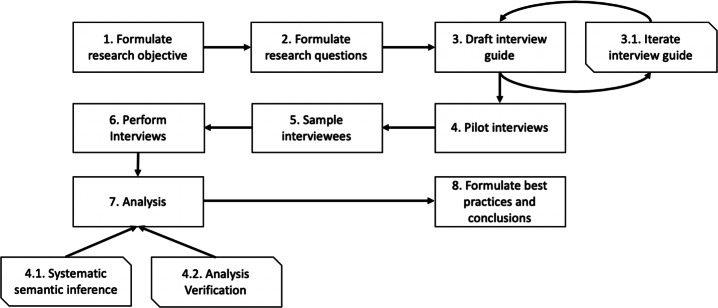
Visualization of the methodology used throughout the study

For reference, Figure 1 provides a visual overview of the research procedure, and its eight steps, conducted in this study. The procedure was orchestrated by the first author, supported by the co-authors who are all practitioners from industry. As such, the study, classified as exploratory but primarily descriptive case study, was performed in co-production between industry and academia. This collaboration was performed within a larger project, where the objective of the study was defined by the industrial practitioners, verified through literature analysis, and then jointly investigated.

In the continuation of this section, each step of the procedure will be described, including sub-activities and other details required to understand how the study was performed. Effort has been spent to describe the study such that it can be replicated. However, we recognize that replication of case studies is inherently challenging. For example, sampling practitioners with identical experiences to our sample is seldom possible. Our measures to mitigate such limitations is discussed further in Section 3.7.

### Formulation of research objective

The objective of this study is to gather real-world experiences from practitioners and industrial projects where MBT, with graphical models, has been adopted, used or abandoned, and transform these experiences into best practices for the technique. This objective was formulated in co-production with the research project’s partners due to a recognized lack of evidence-based guidelines for MBT, in both literature and practice, which cover technical aspects but also present non-technical aspects. In this paper, we divide these aspects as:

- **Technical aspects** - consider MBT development artefacts (e.g. tools, requirements, models or code) and the use of these artefacts for testing or quality assurance.
- **Non-technical aspects** - consider processes (e.g. using models for knowledge sharing, communication to achieve MBT, or when/how to run MBT tests) and organization (e.g. roles associated with MBT or team constellations).

The choice of an interview study with experts is warranted to acquire empirical experiences from real projects. Additionally, we sought experts with multiple years of experience from multiple projects to improve the external validity of the results. Thus, providing the researchers with both in depth details and general practices, used in multiple projects, from which to synthesize conclusions. We sought experiences from successful MBT projects, but since the adoption of any technique or tool in practice is fickle, all experiences were not exclusively from such projects. The reason is because we were also interested in the best practices of abandonment of the technique and believe that lessons learnt from failed projects provide good insights to support success factors. As such, all experiences were of interest but focus for the study was on best practices that relate to MBT success stories.

We recognize that alternative research methodologies could have been used to study the research objective, for instance a tool-focused case study or questionnaire survey. The benefit of a more focused case study would be more in-situ observations of the practices in operation, as well as detailed accounts of challenges. However, since, given the time constraints of our funded project, this methodology would only have given us access to one or a couple of cases. As such, the methodology would not have provided us with the general insights into best practices we were looking for. A more reasonable alternative would have been a questionnaire survey. However, finding a suitably large sample of practitioners was considered a challenge. In addition, a questionnaire would limit the depth of information required to draw evidence-based conclusions. Thus, we concluded that an interview study would be the most suitable to meet our objective.

As stated, we delimit the study to MBT with graphical models due to our industrial partners’ knowledge needs—knowledge about graphical MBT was requested due to currently used tool, e.g. Graphwalker (Zafar et al. 2021). It was also necessary to restrict the study since MBT is a vast research area, including a wide array of tools and techniques, which was perceived infeasible to study given the project’s resources and research team’s contact network. The target audience for the paper is thereby practitioners that seek to adopt, use or avoid abandonment of MBT with graphical models. However, we also hope that the evidence gathered in the paper can provide insights, and complementary results, to research.

### Formulation of research questions

To better understand the MBT life-cycle—from adoption to use to abandonment—and the practitioners’ experiences within this life-cycle, the research objective was broken down into three research questions.

- **RQ1**: What are common experiences that practitioners have when *adopting* MBT, with graphical models, in practice?

This question aims to answer what, if any, experiences that industrial practitioners commonly have during the adoption of MBT. These experiences can be of a technical nature, e.g. connected to tools or frameworks, or concern softer factors, e.g. human- or process-oriented factors. Due to the evidence-based nature of the study, all types of experiences are considered, including challenges, solutions, considerations and prerequisites to successfully adopt the technique.

- **RQ2**: What are common experiences that practitioners have when *using* MBT, with graphical models, in practice?

This question assumes that MBT has been successfully adopted and is being used in a development environment. The question aims to answer what common experiences practitioners have with using MBT in industrial practice, including challenges with using the technique, solutions to said challenges, benefits and drawbacks. As such, aiming to encapsulate MBT usage from a comprehensive perspective, including technical-, human- and process-oriented factors. An additional concept of interest is how MBT compares to other testing techniques or tools.

- **RQ3**: What are common experiences that practitioners have when *abandoning* MBT, with graphical models, in practice?

This question assumes that MBT has been successfully adopted, and possibly used, but practitioners have experienced challenges with the technique that has lead to the decision to abandon—remove or replace—the technique. The question aims to answer what experiences practitioners commonly have during abandonment of the technique. This includes what the warning signs, and causes, for abandonment are, what impact abandonment may have on the development environment and what tools/techniques that can serve to replace MBT after abandonment.

### Drafting the interview guide

#### Development

Guided by the research questions, the interview guide was split into three main parts (one part per research question, i.e. associated with adoption, use or abandonment of MBT) that were individually developed, discussed and improved in iterations through a series of workshops. In the first workshop, the authors used brainstorming to draft an initial set of interview questions. An important aspect when designing the questions was how to elicit the interviewees’ experiences and not their perceptions/assumptions. After the first workshop, the authors worked independently to refine and improve the questions. These improvements were compiled by the lead author in preparation for a second workshop.

In the second workshop, the interview questions were first analyzed from a macro perspective—to verify that they were coherent, consistent and answer the research questions. Second, the language of each question was scrutinized to eliminate ambiguities. After the workshop, a second iteration of independent work was carried out by the authors, and results compiled.

In the third workshop, the interview questions were once more scrutinized and refined. The guide was also expanded to elicit information about the interviewees’ backgrounds. Example questions include interviewees’ years of professional experience in industry, with testing, and with model-based testing. Elicitation of such demographic data is considered best academic practice to to gauge the generalizability of the results (Runeson and Höst 2009). In this study, such information also allowed the researchers to gauge the interviewees’ experiences from successful and unsuccessful projects. As a baseline it was considered that working with MBT for more than a year within one company was a successful project.

The interview guide was written in English but later translated into Swedish—the authors’ native tongue—since most interviews were expected to be held in Sweden.

#### Structure of the interview guide

The final interview guide contains six parts, of which three are the interview questions connected to the research questions.

The first part of the guide is a textual description introducing the study and its purpose. After this description is read to the interviewee, it is explained that all answers are anonymous and that the answers will be disseminated in a way not traceable back to them. Next, the interviewees’ are asked if they agreed to record the interview and told that they have the option to review the study results before they are published, i.e. get early access to this manuscript for review. A clarifying statement is then given about what MBT means and that the study concerns MBT with graphical models. Finally, it is clarified to the interviewees that their empirical experiences are requested rather than their perceptions or theories. The interviewees are asked to clarify when their answers are not based explicitly on their own experiences to separate such results from more theoretical results.

The second part of the guide aims to capture demographic information. This information is required to cluster participants to understand the construct- and external validity of the results. These validity factors are further elaborated upon in Section 3.5.

The third part of the interview guide consists of questions about the interviewees’ experiences with *adoption* of MBT in industrial practice. Hence, interview questions aimed at answering RQ 1. There are six questions—four mandatory and two optional follow-up questions. The questions concern the needs that MBT fulfills, the environmental changes that are required to make MBT work, the roles/stakeholders that shall be involved in the adoption and the challenges with adopting the technique. Together, these questions aim to identify practices associated with MBT success, but also challenges and pitfalls that practitioners should avoid. For the reader’s convenience, the complete interview guide can be found in Appendix A. Note that additional, ad hoc, follow-up questions, not stated in the interview guide, were asked in-situ. These additional questions were not captured in the guide, only in the interview results.

The fourth part of the guide consists of four primary questions regarding the use of MBT in practice, i.e. aimed at answered RQ2. These begin with (1) a question concerning the roles responsible for using MBT, (2) the contextual needs and circumstances that should be considered when choosing MBT for testing, (3) the guidelines for successful application of MBT, and (4) the functional and extrafunctional (non-functional) attributes that can be tested using MBT. Question (3), question 7 in the guide, regarding guidelines for using MBT is divided into ten sub-questions. These questions provided the largest contribution to the study results, and for the readers’ convenience we have classified them below as *technical -* **T**, *organizational - ***O**, or *process -* **P** oriented. These sub-questions concern:

1. What input shall be used to develop/use MBT tests (**T**),
2. How shall test functionality be divided between the graphical test model and MBT test code used to drive the model execution (**T**),
3. How much time shall be spent on modelling compared to test code development (**P**),
4. How shall development of models/code be divided between different roles (**O**),
5. How/when shall model-based tests be maintained (**P**),
6. How often shall MBT tests be run (**P**),
7. How shall models/code be quality assured (**P**),
8. How are models and code effectively reused (**T**),
9. How are test scenarios efficiently recreated when MBT tests fail (**T**), and
10. How is output most effectively used from MBT tests (**P**).

Combined, these questions seek to identify practices associated with successful use of MBT but also challenges, or pitfalls, that practitioners should avoid. In particular, the listed sub-questions elicit practices that the interviewees’ consider favorable, based on their own experience. Note that the questions are stated agnostic of tools and type of graphical models used. The questions also concern process-, organizational- and other environmental factors—i.e. non technical factors not well-described in previous research.

The fifth part of the interview guide are questions concerning the abandonment of MBT and reasons for abandonment, i.e. aimed to answer RQ3. There are five questions in total, four mandatory and one optional follow-up question. These questions concern the reasons and warning signs that MBT should/will be abandoned, what effects abandonment has on daily work, what tools/techniques that best replace MBT after abandonment and finally what the interviewees would have done differently to succeed better if they got to re-introduce MBT in a context where MBT has previously been abandoned. Due to the study’s objective of capturing best practices, less emphasis was placed on the abandonment of the technique since such results are inherently associated with failed projects.

The aim of these questions is to capture aspects that can lead to the abandonment of MBT and possibly ways of preventing them. Also, assuming that the technique is abandoned, what can be done, if anything, to fill the gap that MBT leaves behind in terms of testing.

In the sixth, and final, part of the interview guide the interviewees are asked if they have any additional comments that they feel have not been covered by the interview questions. This question gives the interviewees the chance to complement the interview if they feel (1) the interview instrument was not complete or (2) if there are unique factors in the interviewees’ context that required special consideration.

Combined, the six parts of the questionnaire cover the entire life-cycle of MBT adoption, use and abandonment, eliciting experiences and other observations from the interviewees. These results are then used as input for further analysis, discussed in Section 3.7.

#### Statement on validity of the guide

The interview guide development was driven by the lead author who has previously published several interview studies in the area of software testing. The questionnaire was drafted and refined through academic best practices with questions based on the authors’ combined empirical and industrial experience (Runeson and Höst 2009; Baskarada 2014). Whilst this domain knowledge helps improve the questions’ construct validity, we cannot exclude biases caused by questions being poorly formulated or that a more favorable question may have unintentionally been left out. To address these possible threats we designed a deep questionnaire guide, chose a semi-structured interview design to enable follow-up questions and gave interviewees the possibility to give additional information at the end of the interviews.

### Piloting the interview guide

Interview pilots are used to test that the interview protocol (1) will be correctly understood by the interviewees and (2) if the interviewees’ answers fit the researchers’ expectations. Two pilot interviews were carried out in sequence during the study, with improvements of the first pilot carried over and tested in the second pilot. The first interview was held in Swedish with one of the authors of this paper who had not been involved in the final refinement stages of the questionnaire. This person is considered an MBT expert and well knowledgeable of the study’s research objective. As such, able to comment upon the domain validity of the questions, if they were ambiguous, and their connection to the research objective. Additionally, the time required to run the interview could be tested. The first pilot only resulted in minor, mainly linguistic, changes to the interview guide and it was determined that the interview could be performed in under 60 minutes.

The second pilot was performed with the English interview guide after being updated according to the feedback of the first pilot. It was conducted with an English-speaking interviewee, external to the research team, which could therefore give feedback on things the team may have overlooked. The second pilot confirmed that the interviewee found the questions easy to understand and answer. Further analysis of the interview results showed semantic similarity in key areas to the answers of the first interview, implying correctness of the guide.

Because of the pilots’ success, the decision was taken to proceed with the interviews. Additionally, since no changes were required to the interview guide that affected the semantic meaning of the questions, the pilot interviews were included in the final data set.^2^

### Sampling interviewees

The sample frame for the interviews was intentionally delimited to software engineers with experience of using MBT, with graphical models, for testing software-intensive systems. This decision explicitly excludes embedded systems development, e.g. the automotive or aerospace domain, where MBT is also used (Villalobos-Arias et al. 2019). The motivation for this delimitation, as stated, were the needs of our industrial partners, resource limitations but also, based on our experiences and literature review, that evidence-based studies are limited in the area of software focused products.

However, despite this delimitation, the sample frame is still vast and therefore infeasible to comprehensively cover in the study due to resource constraints. This constraint is also one of the rationales for why the study focuses on guidelines for MBT with graphical models and not MBT in general. Regardless, finding experts in industry, which are also willing and able to participate, is a time-consuming process. The interviewees for these interviews were sampled either from the first or second tier of the authors’ personal contact network—contacts or contacts of contacts—or were practitioners who volunteered through the Graphwalker tool’s forum. However, to get a suitably large sample of interviewees, with suitable experiences, we relied on convenience sampling and focused on performing as many interviews as possible. Hence, we did not screen, nor exclude, interviewees. Instead, screening was performed on the interview results, judging demographic data as well as coherency of interviewees’ responses.

Due to the chosen sampling approach, no random inclusion/exclusion of participants was possible to control the sample. However, analysis of the interviewees (N = 17) shows that it includes:

1. Engineers with 8 to 30 years of **industrial experience** (average 19.5 years, standard deviation 8.4 years),
2. Engineers with 5 to 30 years of **testing experience** (average 14.7 years, standard deviation 7,8 years),
3. Engineers with 1 to 16 years of **MBT experience** (average 6.4 years, standard deviation 6.3 years),
4. Engineers with varying roles, including MBT test developers, testers, team managers, QA engineers and MBT tool developers. As such, individuals with varying operational roles, able to provide experiences as developers, QA engineers, champions and project managers within/for MBT projects, Engineers from Canada, Denmark, Germany, Sweden, United Kingdom, and the United States,
5. Engineers from ten companies—most with experiences from several companies or projects—experienced with testing and developing leisure applications (e.g. music streaming), banking systems, insurance systems, finance systems, testing tools, MBT tools, front-ends for large-scale systems, communication/telecom systems, governmental systems as well as consultancy within quality assurance,
6. Most of the interviewees had experiences from longer consecutively running projects, i.e. projects longer than 1 year. As such, their experience enabled elicitation of best practices from successful MBT projects,
7. Nine of the interviewees were previously known by one or more of the authors, the remaining eight were previously unknown to all of the authors.

We conclude that this set of interviewees span a representative range of domains, companies and platforms—although not comprehensive—to cover most software intensive systems. The sample also represents experiences from ≫ 17 projects. Thus, enabling elicitation of results with high external validity. In addition, we note that most practitioners had been part of at least one project with longer running time (> 1 year), implying project success. For instance, MBT was used for over ten years at a music-streaming software company, in which several interviewees worked/had worked. Other examples were two 3 year MBT projects within the governmental and financial technology sectors. We do however stress that no explicit question, nor criteria for project success, were given to the interviewees. The conclusion of interviewees’ experiences of successful projects is however strengthened by the interviewees’, on average, 6.4 years of industrial experience with the technique. We recognize that this metric is influenced by the number of projects each interviewee has experiences from. This information was not explicitly elicited, but through analysis of the interviews we found that most only mentioned experiences from a maximum of two to three projects. Thus, allowing us to conclude that most projects were longer than the set threshold for success of 1 year.

Software industries that are not represented are the aforementioned automotive industry but also the gaming industry where MBT and model based engineering (MBE) is sometimes used. However, due to the inherent commonalities of modern software development, we expect the adoption, use and abandonment of MBT to be similar. Additionally, the results are synthesized, triangulated (Runeson and Höst 2009; Baskarada 2014) from several sources, and presented on a level of abstraction which should increase the probability of the results being of benefit to non-represented domains.

### Conducting the interviews

The interviews were conducted during 2020, at the peak of the COVID-19 pandemic. Therefore, all interviews were conducted online, using either the communication platforms Zoom or Microsoft Teams. The audio of each interview was recorded and detailed notes were taken, close to real-time transcription, by the interviewer. After the interviews, the recordings were used to complement the interview notes, adding details as required during validation of the transcriptions.

All interviews were carried out by the lead author and were planned to be 60 minutes each, estimately split accordingly:

1. (Part 1) Introduction to study, interview, clarification of concepts and request to record the interview (5 minutes),
2. (Part 2) Elicit interviewee background information (5 minutes),
3. (Part 3) Elicit the interviewee’s experiences with the adoption of MBT in industrial practice (15 minutes),
4. (Part 4) Elicit the interviewee’s experience with the use of MBT in industrial practice (20 minutes),
5. (Part 5) Elicit the interviewee’s experience with the abandonment of MBT in industrial practice (10 minutes),
6. (Part 6) Elicit other concerns the interviewee thought had not been covered in the interview (5 minutes).

However, since many of the interviewees were eager to share their experiences, most interviews ran longer than 60 minutes. The average time for the interviews was clocked to 74 minutes (median 66 minutes) with a standard deviation of 23 minutes. The shortest interview was 54 minutes and the longest 137 minutes, measured from the audio recordings.

The willingness of the interviewees to share their experiences made it possible to ask in-depth follow-up questions to explore key concerns. This willingness to contribute also reflects the interviewees’ knowledge/experience, passion for the technique.

The interviewees’ eagerness was however identified as a threat; interviewees may overstate the benefits, or understate the drawbacks, of the technique. To mitigate this threat, the interviewer took a critical stance towards the interviewees’ statements. In particular, asking interviewees to elaborate when their statements differed from other interviewees or the literature. Additionally, follow-up questions were asked throughout the interviews to challenge, or confirm, the coherency of each interviewee’s statements. To further verify the validity of the interviewees’ statements, the results were scrutinized during the analysis phase (reported in more detail in Section 3.7) for consistency and coherency.

It was made clear to the interviewees that we sought their real world experiences, and practices they had applied in actual projects. We also asked the interviewees to clarify if their statements were theoretical. By this practice we argue that we could elicit practices that are verified, through empirical use, in practice. As such, due to our strict evidence-based analysis, the synthesized practices are also considered valid for industrial use. The list of practices was also verified by the practitioners that took part in the study, discussed further in Section 3.8.

The results of the interviews were stored in audio file recordings and transcripts in digital documents (Microsoft Word). These files were structured question by question and were the primary source of data for the analysis. During the analysis, the audio recordings were only used when exact quotes from the interviewees were required.

### Analysis

To achieve the study goal of identifying general best practice guidelines for the adoption, use and abandonment of MBT, an evidence-based analysis approach was employed.

**Fig. 2:**
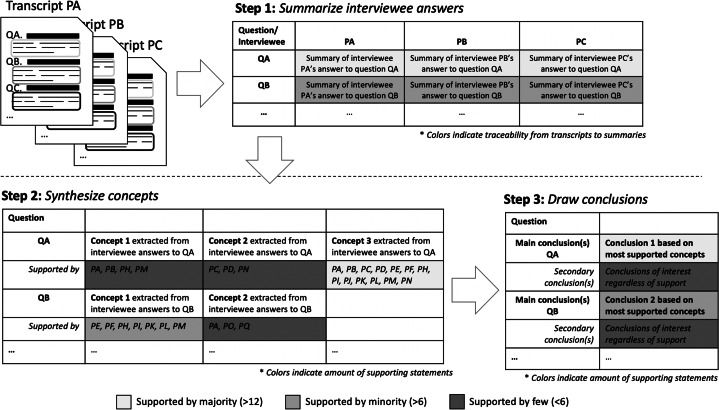
Visualization of the three step procedure used to analyze the interview answers and quantify them based on supporting statements from the interviewees. **Note:** *Color coding in the top part of the figure indicates traceability to interview transcripts. Colors in the lower part indicates amount of interviewee support*

The analysis procedure was divided into three steps, as visualized in Figure 2, taking the interview transcripts as input. In the first step, the interview transcripts were reviewed interview-by-interview and question-by-question to extract summaries of each answer. The summaries were collected in an Excel sheet, as shown in Step 1 of the figure, with the interview questions in the first column and the summaries per interviewee in succeeding columns. When summarizing the answers, care was taken to retain the semantics of each interviewee’s answer and include as much information as possible. This practice was conducted by the leading author but reviewed and verified for suitability by the other authors. Note that information exclusion was kept to a minimum at this stage, i.e., all answers were summarized. This is because this step of the analysis served to transform the interview data to make it easier to overview. As such, it did not serve to sample results for the synthesis or to draw conclusions. Instead, this step was performed to enable further analysis with lesser need to use the raw interview transcripts. This practice also made it easier to identify parts of answers to one question that were connected to other interview questions. When such answers were found, they were also connected to the correct questionnaire questions. This was done by copying the part of the answer related to another question to the row of the Excel sheet related to the correct question.

In the second step of the analysis, the summaries were analyzed row by row in the Excel sheet to synthesize the concepts of interest—mentioned by several interviewees—based on the semantics of each statement. The identified concepts were written down incrementally together with a label representing each interviewee, P_*X*_, which gave a supporting statement to the concept (See Step 2 in Figure 2), denoted by P_*X*_ ∈*P*_*Xs*_, where P_*Xs*_ are all interviewees. In a few cases, when the hypothetical person P$_{X}^{\prime }$ provided complementary information to the original contributor P_*X*_, the concept was augmented by appending additional detail. However, at this stage, care was taken to ensure that no information was added to the concept that would contradict previous supporting statements. Instead, the plan was to add a new concept with contradicting statements if such was found. Additionally, when adding supporting interviewees to concepts, care was taken to ensure that the supporting statement was actually supporting the original concept. In cases where there was doubt, the original transcripts were reviewed to clarify the intent of the interviewees’ statements. However, in none of the doubtful cases did review of the original transcripts/audio recordings change the outcome. This outcome also provides support that the summaries developed in Step 1 of the analysis were of suitable quality and detail.

Once all the concepts had been synthesized, the table of data was reviewed and supporting interviewees, for each concept, counted. The counting served to identify which concepts were the most supported. To quantify support, the following, rough, guideline was considered:

- 1-6 (0-35%) interviewees - Concept experienced by a *few* of the interviewees,
- 7-12 (36-70%) interviewees - Concept experienced by a *significant proportion* of the interviewees,
- 13-17 (71-100%) interviewees - Concept experienced by a *majority* of the interviewees,

In the third step, the synthesized concepts were analyzed row by row to draw conclusions associated with each interview question. This was done by looking at the concepts with the most support from the interviewees and writing statements containing the identified concepts. Concepts only supported by a few interviewees were then merged to form secondary conclusions. The justification for adding the secondary conclusions is that answers to interview questions still relate to the same question. Hence, many of the answers, albeit not semantically similar enough to be combined, could still be verified by other results. Alternatively, they could be verified using literature or through logical inference.

In Figure 2, under Step 3, the appearance of the resulting table of conclusions, with main and secondary conclusions, is shown. In this table, the conclusions were also color coded. In the example, the main conclusion for Question A (QA) is perceived to have more support than the main conclusion of of Question B (QB), whilst the secondary conclusions of both have less support and are therefore darker.

At this step of the analysis, to verify the correctness of the results (as shown in Figure 2), and to remove any selection bias, the authors were each given four unique interview transcripts to review. The instructions for the review was to go through each interview, write down observations of interest and then do a synthesis of the observations. Hence, a light-weight version of the more systematic analysis approach presented in Figure 2. These independent reviews were then sent to the first author who compared the individual reviews to the complete analysis. The validity of this verification approach is justified by the authors’ large domain knowledge and expertise in MBT. Although the latter presents some potential for selection bias, this is not considered an issue since the first author has considerably less domain experience than the other authors.

The result of the conducted comparison was that the larger analysis (visualized in Figure 2) had captured *all* observations the other authors had listed.

### Formulation and verification of best practices

The analysis resulted in 13 high-level conclusions that were transformed into 23 best-practices for MBT. The transformation was done by the research team by scrutinizing the conclusions and breaking them up into tangible components. Each component was then discussed and reformulated into a practice, or suggestion, relying on the team’s expert knowledge of MBT and software engineering in industry. Explicit practices mentioned in the interviews, as well as academic references, were also used as support or inspiration. To retain traceability between the practices and the conclusions, the practices are presented in Tables 2, 3 and 4, and connected to the conclusions. Each practice is also discussed under sections titled as the conclusions (See Section 4). The rationale for this choice was that it provides a suitable narrative for the reader, which also complements the summaries presented in the aforementioned tables. To strengthen the discussion further, example quotes from the interviewees have also been added.

The number of practices, 23—i.e. more than one suggested practice per interviewee—is perceived to the outcome of the interview questions, which elicit practices of the adoption, use and abandonment of MBT. These questions, in combination with the deep interview design, enabled us to identify and triangulate a rich set of results. The outcome is in any case evidence-driven and the formulated conclusions and practices are thereby completely based on the interviewees statements. This choice has been made to maximize the industrial validity of the results.

However, since the practices were defined based on expert judgement by the research team, care was taken to externally verify the results. For the verification, the lists of conclusions and best practices were sent to the interviewees for feedback. Out of 17 interviewees, five had minor feedback on the conclusions and best-practices but all interviewees agreed with the results themselves. Worth noting is that all interviewees agreed with the results despite of the interviewees’ varying answers to the interview questions. This observation implies that the synthesized results are on a suitable level of abstraction to capture all the interviewees’ experiences. An alternative explanation is that the interviewees chose not to voice their objections against the results, or disagreed with them but not enough to reject them. However, given the interviewees eagerness to contribute to the study, we expect this to be unlikely.

As a secondary form of verification, the results of the study have been disseminated in the authors’ companies. Feedback from these instances have been positive, further providing us with confidence of the results’ validity.

## Results

Through analysis of the interview results, 13 conclusions are drawn from which 23 best practices are derived for the adoption, use and abandonment of MBT in practice. These practices are directly synthesized, throught triangulation, of the interviewees’ statements using the analysis procedure presented in Section 3.7. This section will present the conclusions, split according to the research questions, i.e. results referring to the adoption (RQ1), usage (RQ2) and abandonment (RQ3) of MBT in practice. A summary of the conclusions is presented in Table 1. The table also presents if a conclusion has been classified as technically-, process- or organizationally-oriented.

**Table 1:** Summary of the evidence-based conclusions drawn during the study about the adoption, usage and abandonment of model-based testing in industrial practice

Phase	Conclusion	#	Description	T/P/O
**Adoption**	Level of abstraction	1	The level of model abstraction must be considered from early adoption and individuals with aptitude, or training, in model abstraction, and with domain knwoledge, be put in charge of the modelling.	**O**
	Mindset	2	To optimize effectiveness and efficiency of model-based testing, an organizational mindset should be adopted that fosters close collaboration between individuals with domain- and technical knowledge.	**O**
	Guidelines	3	Guidelines for model and test code management shall be established, preferrably based on existing guidelines for source code management.	**P**
	Test purpose	4	Model-based testing is best suited for longer and non-trivial test scenarios, e.g. with multiple alternative scenarios, where fuzzing can also be utilized.	**T**
	Re-execution	5	MBT tools should support repeatable execution of generated tests, either directly from the model or via test execution logs.	**T**
**Usage**	“Good” models	6	Models should, preferably, model system behavior, be concise and simple, fit-for-purpose (i.e. suitable for the level of test and its purpose) and readable by all stakeholders. They should also be based on requirements, and although scenario-based requirements are preferred, this is not a strict requirement.	**T**
	Verifying MBT tests	7	Model and test code verification shall be performed with the same rigor as source code. Reviews is a recommended approach but also static code analysis and mutation testing can be applied.	**P**
	Maintenance	8	Proactive maintenance, at requirements’ change, is recommended but reactive, rapid and frequent, maintenance due to test failures, similar to other automated testing, shall be the norm.	**P**
	Test logic	9	The logic that governs the test scenarios shall be modelled, whilst explicit test logic (e.g. Assertions) shall be defined in the MBT test code.	**T**
	Return on investment	10	Model-based tests shall be run often, recommended two to three times per day with a minimum recommendation of nightly runs.	**P**
	Functional vs Non-functional tests	11	Model-based testing is best utilized for functional testing, but can be utilized for non-functional tests such as performance tests.	**T**
**Abandonment**	Causes of abandonment	12	Knowledge of the model-based testing solution must be shared within the organisation.	**P**
	Warning signs	13	Warnings signs, indicative of the struggles with model-based testing, include evaluation or other testing techniques and sudden rise in maintenance costs.	**P**

T - technical, P - process and O - organization

### RQ1: Adoption of MBT

#### Levels of abstraction

MBT requires engineers to define the level of abstraction to model the tests, normally corresponding to the testing needs of the SUT. This level, for instance system level, governs the look and feel and the amount of information detail required in the test models. As a side effect, this level also places requirements on the MBT test code. For example, if the models are on high level of abstraction, each node will naturally be associated with more code.

However, as stated by the interviewees, individuals’ aptitude for abstraction/concretization of information varies—some people find it difficult to go from one level of abstraction to another—thereby affecting the individual’s natural ability to create MBT tests on the correct level of abstraction. An example from a telecom company was expressed as: “*So many times people said: I know what I am doing, Modelling is a waste of time, let me code. One of the significant challenges is to find people that have the aptitude and inclination to abstract problems.”*. Although this challenge is logical, in the same way that different people have varying aptitude for music or art, it is a commonly overlooked phenomenon and often not considered when appointing responsibilities.

Additionally, the results show that individuals with different organizational roles tend to model behavior in different ways. For example, while developers often model tests on a more technical level of abstraction (e.g. including more technical details), domain experts generally prefer to instead model on a user level of abstraction. As stated by an interviewee, “*...the modeler should still have a testing background. The test code engineers don’t have to have a testing background but it’s beneficial because they would know what they’re doing. The modellers however need to have a strong foundation about decisions, coverage, etc., to create good models.”*. This observation also correlates with individuals’ perception of the amount of effort that shall be spent on, and importance of, formulating test models versus writing test code. While domain experts tend to favor placing test functionality in the model rather than MBT test code, people with a technical persuasion tend to think the opposite.

Regardless, the interviewees’ generally highlight modelling to require more effort than code. Therefore—although collaboration among stakeholders is important to the success of MBT—domain experts are generally the most suitable to create models. In contrast, developers are generally more suitable to perform the technical implementation of test code. This conclusion is logical since it makes the most use of individuals’ expertise where it is most applicable.

#### Mindset

MBT efficiency and effectiveness is reliant on an organisation’s ability to use models to communicate and collaborate among stakeholders; “*Also [MBT has] a benefit of combining domain experts and technical people. If we are talking about the process of building a MBT framework then everyone should be able to contribute it.”*, which was later followed up with “*The more people that use the model the better, you shouldn’t have knowledge silos.*”. This way-of-working requires a different mindset from traditional automated testing where tests, to a great extent, can be written and performed in isolation by testers or developers. As state by one of the interviewees, “*However, for someone that doesn’t have this [MBT] mindset, there are barriers to get into how to work with MBT. ...it’s not a 180 switch... But some people might not realize they are thinking about models.* In particular, this mindset shall foster communication between individuals with domain- and technical knowledge; “*To visualize the tests just makes it easier to bring it to life.”*, “*[MBT] Covered all testing needs, sanity/regression and exploratory tests. Very easy to visualize the scenarios for both testers and managers. We can visualize scenarios for domain experts.*”, and “*Especially if you have stakeholders that are interested in what is tested but not the technical aspects, then it is suitable to show a model that is simple to understand*”. MBT can be performed in isolation but then loses many of its core benefits (e.g. early defect identification/prevention) provided by cross-organizational modelling. Isolated modelling is also more prone to erroneous test generation since important domain information may be lacking in the models.

#### Guidelines

Test code, similar to source code, shall be well structured, documented and managed with the same rigor as source code to not degrade over time. Although MBT provides inherent architectural support, the test code may still, for some components, be intricate and therefore needs to follow suitable coding best practices. For instance, the interviewees expressed the need for both guidelines and coding conventions; “*One thing we did at [a large operating system’s manufacturer] two-thirds into it [the project] was to write design guidelines for the top and bottom [model] layers.*” and “*But also, it comes down naming conventions. What I’ve seen outside MBT is that people rewrite stuff because they couldn’t find stuff.*”. Furthermore, for MBT benefits of reuse to work, prerequisites and post-conditions of state transitions need to be well defined. Failure to achieve these were by the interviewees stated as detrimental to the cost of reuse of existing components.

Aligning MBT guidelines with existing guidelines, environments and ways-of-working is also expressed by the interviewees to be a success factor. Similarly, as discussed, all changes to the ways-of-working, associated with the change in mindset, shall be documented as guidelines. The interviewees have experienced different degrees of documentation but as a bare minimum, contact surfaces, i.e. key stakeholders that act as communication interfaces between groups, shall be communicated to everyone working with MBT.

#### Test purpose

MBT, unlike many other testing techniques, can be used for more than one test purpose. As an automated technique, MBT is commonly used for regression testing. However, as expressed by the interviewees, MBT is also a suitable tool for random/fuzz testing. This was for example expressed by interviewees as, “*[MBT] It fulfills the regression testing purpose as well but it’s more searching tests... ...There are examples where such tests find good bugs.*” and “*Often used it [MBT] in GUI testing circumstances, to test flows. Ran random variants, random walk-throughs through the GUI when you don’t want the exact same flow. Others have used the same approach, a little, for APIs...*”. A conclusion drawn from the results is that MBT is most suited in environments that require, or have a need for, such scenario-based or random tests. Interviewees expressed that MBT was favorable when; “*If I have a system where many parameters are set and there are many combinations, then MBT is good.*”, “*...if you have MBT in your tool-box, and it’s something you use, then it’s for scenario-based tests, then it is suitable.*” In addition, the interviewees stated that in projects where pure regression testing has been used, the costs of MBT may outweigh its benefits, with potential negative impact on the approach return on investment.

Similarly, because of the scenario-based nature of MBT, testing on higher level of abstractions are considered more favorable, i.e. user scenario-based tests, then lower level tests. Lower level tests were stated to have a tendency to not include enough combinatorial complexity for MBT to provide a significant benefit over other automated techniques; “*I would use MBT on the highest test level, E2E or exploratory testing. On lower levels I would not use MBT as a first alternative*”. Alternatively, the functionality is state-less; “*If it’s state-less, e.g. [testing] the HTTP protocol, then MBT isn’t necessarily good. On the other hand, if the system builds on HTTP, which pulls states, and you have a scenario with switches, then MBT can have value.*”.

#### Re-execution

The most common way of capturing the outcome of MBT tests is through automatically generated log-files. In addition to capturing test results, these files capture test scenarios so they can be re-executed. The captured scenarios allow the tests to be used for regression testing, to verify that treatments of faults have had effect or to replicate failures to find their root cause.

However, the interviewees stress that not all MBT tools support automated re-execution of random model traversal of test sequences. As such, much of the re-execution is reported to be manual in practice and therefore costly since randomly created test sequences can be long (include many steps); “*This [re-execution] is a challenge but you have to think about it when you code, to write out the steps of the test execution... ...Troubleshooting was manual.*”, “*Also manual replication in several cases since it can be difficult to pause the model and debug at the same time.*” and “*The best is to have a lot of good logs. Use detective work and manual work, if possible, to recreate errors.*”. Automated re-execution is therefore preferred and the interviewees state that they, themselves, have added such functionality to existing tools. This was achieved either by producing executable log-files or by creating tools that can parse, interpret, and execute, such files.

Adding such functionality is thereby possible, but requires significant investment by the adopting organisation. As a result, the interviewees claim that automated re-execution of generated test scenarios is considered a challenge for MBT.

#### Summary for RQ1

From the results we note that the conclusions capture a wide range of areas associated with the adoption of the technique. First, the level of abstraction of models also relate to the *individual developer’s* aptitude for abstraction. Second, the MBT mindset is discussed, which relates to the *organization’s ways-of-working* and thinking about the technique. Third, guidelines are important which is a *process perspective* of how to align and document ways-of-working. Fourth, the test purpose is considered, which is a broader concept applicable both to the *individual developer and the organisation*. Finally, re-execution of random model traversal is a *technical challenge*. Combined these results highlight that adoption of MBT requires much more than just technical solutions and that varying experiences, with each of these factors, influence the success of MBT adoption within an organisation.

### RQ2: Usage of MBT

#### “Good” models

MBT tests shall be modelled based on the SUT’s requirements. Additionally, although some interviewees propose to model tests, most interviewees instead propose to model the SUT’s behavior. While these two types of modelling schemes may seem equivalent, there are some important distinctions. Models of tests are generally more focused on explicit scenarios and focused on stimulating faults, or document previously found faults. Models of behavior instead aim to capture the intended behavior of the SUT as specified by the requirements. Hence, a more general description of the SUT compared to explicit test scenarios. Exampels expressed by the interviewees include, “*To use models [to model SUT behavior] has been good to find problems and ambiguities in requirements.*” and “*The model doesn’t show how the system shall work but rather examples of how the system can be used.*”.

To achieve this, the interviewees stress the need to model on a suitable level of abstraction. They also stated that it is important to keep the test sequences as short and simple as possible to make the models easy to overview and scale.

Some characteristics of a “good” model, stated by the interviewees, are: (1) fit-for-purpose (i.e. defined on a suitable level of abstraction and align with the test’s purpose), (2) easily readable by all stakeholders, and (3) communicate system behavior and valid inputs for testing, but more characteristics can considered. However, how to satisfy these criteria in practice depends on the context and the domain. For instance, fulfilling (1) when testing a system’s Application Programming Interface (API) may require more detailed models than testing the same system’s Graphical User Interface (GUI).

Inputs used for MBT test development do not have any special prerequisites (e.g. specific types of software requirements, SUT architectural or interface specifications) compared to other automated testing techniques. However, scenario-based requirements (e.g. use cases or requirements written with Gherkin (North 2010) syntax) were mentioned by the interviewees as beneficial since they generally describe system states and state transitions. Hence, information that is well suited for modelling. Models can, as stated, be developed on other requirements, but may require more assumptions to be made by the developer. Faulty assumptions can lead to higher cost, but also frustration between the stakeholders.

#### Verifying MBT tests

MBT tests shall, according to the interviewees, be verified in the same way as source code or other automated test code. A particularly beneficial approach is to have stakeholders periodically review both the models and the test code derived from, or used to run, the models. As expressed by some the interviewees, “*Reviews, this [MBT test code] doesn’t differ from other programming.*”, “*Especially reviews, peer review, during development. Depending on the organization, you can also include reviews from stakeholders outside the team, e.g. a requirements engineer.*” and “*Models and [MBT test] code can always be reviewed. However, it shall be reviewed by the right people, both requirements engineers and testers; is it a good test?*”. This enables both domain and technical experts to identify faults early in the development process, which is beneficial to the quality of both the SUT and tests. This is connected to the previous conclusions about “good” models and helps mitigate faults caused by assumptions.

Automated verification approaches can also be considered, including static code analysis (Habib and Pradel 2018) and mutation testing (Offutt et al. 1996); “*[In regards to quality assurance of MBT models and MBT test code] I would say a combination of reviews and static code analysis*”, “*For [MBT test] code you can do the same as with any other code, e.g. use SonarQube (Static code analysis tool)*” and “*If you really want to test your tests you could involve a bit of mutation testing. We have a model that we’ve built and inject a known fault and we know it’s there and run through our model and kill the mutant*”. Static code analysis served to remove code smells in the test code or code architecture, whilst mutation testing was utilized to expand the models with additional test sequences or test data. However, as also expressed by the interviewees, both of these practices are uncommon, nor stated as a general recommendation due to their cost. Instead, the aforementioned reviews were considered as the best approach.

#### Maintenance

The interviewees’ experience is that MBT tests require frequent maintenance, which can be applied in a reactive fashion, after tests fails, or proactive, after requirements change. Both practices, as for other test techniques, serve to mitigate further degradation of the test suite. As expressed by interviewees, “*A proactive solution [to maintenance] is better. As a developer that has written the tests, I should have them with me [working] in my backpack all the time.*”, “*If you include the models as requirements the management becomes easier and then you work more proactively than if you fix it later.*” and “*Proactive maintenance is always better! A risk otherwise is that you miss to add something.*”.

Proactive maintenance is generally recommended but experienced by the interviewees to be used more seldom in practice due to its additional overhead costs and requirements for traceability matrixes between requirements and models; “*The ideal is that you want to be proactive but very few companies seem to be there. If a company’s culture is such that you have a high amount of communication and people can work together, then you can be proactive.*”. Instead, the more common approach is that MBT tests are evaluated through execution, after which failing test sequences, and/or test codes, are reactively maintained. While the reactive approach is generally considered good enough, a danger lies in maintenance cost piling up and lead to degradation of the tests over time. As expressed by one interviewee, “*If you have test runs that start to indicate failure, you have to analyze and fix the source code or the [MBT] test code. It’s a problem area that test code doesn’t receive enough love and get left alone to start to rot and becomes troublesome to fix.*”. The interviewees stated that they experienced this phenomenon of cost pile-up less in projects that practice proactive maintenance.

#### Test logic

For this study, an assumption is made that MBT test sequences are split between the MBT model and the MBT test code. The interviews provide inconclusive results regarding how much test logic that shall be placed in the MBT models versus the test code. Hence, while some interviewees stated that the majority of the test functionality, upwards of 90 percent, shall be placed in the test model, others said the complete opposite. This result can be attributed to, as previously stated, the roles of the interviewees.

However, the general consensus among the interviewees is that logic that governs the scenario execution shall be defined in the model, whilst explicit test logic (e.g. functionality from supporting libraries and assertions) shall be defined in the test code. As stated by a few interviewees, “*If possible, all [logic] shall be in the [MBT test] code, that’s the ground rule. The model is not meant for coders. Logic in models can, for example, be how often paths are taken. The type of logic a non-technical person can find logical. Logic in the models govern test flows.*”, “*I am leaning towards having more logic in the code than in the models to keep the models simple. Control flows in the model, technical logic in the [MBT test] code.*”, and “*An extended state machine has a lot of possibilities and challenges. You should let models govern the [‡ est] flows. For instance, the graph decides that you shall log-in as an admin, but the [MBT test] code decides what privileges you log in with.*”.

#### Return on Investment (ROI)

MBT tests shall according to the interviewees be executed as frequently as possible, preferably as part of a continuous integration (CI) environment. However, due to the costs associated with the random model traversal and test execution, which in extension is a challenge for CI pipeline integration, only 2-3 times a day is considered a realistic expectation. As expressed by interviewees, “*Not every commit, every night. You want the tests to be more exploratory, let them run for a few hours during the night instead of too quickly. The slowness [slow execution] is important.*”, “*As often as possible, at every change if there is time, otherwise at least once a day. Important to get measurements on if the tests are stable.*” and “*The norm is to run them when you commit something... ...You run just the API tests at that time and run the GUI tests at night.*”. The minimum recommendation, based on the interviewees’ experiences, is to run the models at least nightly; “*As often you can. Every night as a minimum but preferably more often.*”. Less than nightly executions do not to provide frequent enough feedback to justify the development costs, nor provide quick enough feedback to keep maintenance efforts to a minimum.

#### Functional vs non-functional tests

The interviewees had little experience with non-functional (quality requirements) testing with MBT, except for performance testing. For example, two interviewees said, “*Robustness tests, memory leaks that only occur over time. Then [to find these] you want realistic loads [on the SUT], which you get with MBT.*” and “*I personally use it to identify response times and memory leaks.*”. Another example statement was, “*I have used it for functional testing and during performance tests. The model-based tests ran as functional tests, but load, from load tests, set the server under pressure at the same time as the model-based tests were run to check that the functionality worked when there is max load on the servers.*”. The interviewees did however explain how MBT could, theoretically, be used for other types of non-functional requirements, for instance security. However, since these practices are theoretical, they are not discussed in further detail.

#### Summary for RQ2

From the results we once more note a variety of impact areas of each of the conclusions. First, “good” models refer to the *individual’s knowledge* of how to model the SUT and if tests are modelled or the SUT’s intended behavior. Second, how to verify the correctness of MBT tests both concerns *organizational practices*, i.e. through reviews, or a *technical aspects*, e.g. through automated tests. Third, how to maintain the tests is a *process and management consideration*, considering when and how to maintain the tests. Fourth, how to divide test logic between models and test code is a *team-level concern* that also requires collaboration between stakeholders. Fifth, return on investment is an *organizational* question, where failure to achieve return on investment can be a cause of the technique’s abandonment. Finally, using MBT for testing SUT quality characteristics—non-functional requirements—is possible, but a underutilized area of the technique in industrial practice and thus a *technical concern*. Combined, these results once more showcase the plethora of different factors that go into the successful usage of the technique in practice.

### RQ3: Abandonment of MBT

#### Causes of abandonment

The most common cause of MBT abandonment was expressed by the interviewees as caused by champions, or people with knowledge about the technique and its implementation, leaving the organization. Thus, resulting in the knowledge of how to use the MBT tests being lost. As expressed by one interviewee, “*It is often because the people with competence disappear. The people that are left are not knowledgable in it [MBT], neither understand or burn for it. Then it dies out. You have to have a champion for it*”. Training, documentation and knowledge sharing about the tests and technique are therefore important activities that require support from the entire organisation. As expressed by interviewees, “*You abandon MBT because you don’t understand what you introduced and did it poorly. You think you can solve it with another solution [tool or approach]. Nothing changes due to the abandonment. The solution to the problem is rather to understand the problem. Competence and politics. [MBT] is abandoned due to non-technical reasons.*”, “*A reason I have experienced is that you try to invent, introduce, new testing tools. New ways of doing things come from the side all the time. You then feel that the developers are not comfortable. To mitigate this you have to be able to show the benefits of MBT och what value you can get from it.*” and “*The reason in the both projects [from which the interviewee had experience of abandonment] was too much maintenance. It was difficult to keep up with development.*”. This observation relates to the required change in mindset, where MBT is more of an organisational responsibility than just the responsibility of testers.

#### Warning signs

If individuals or teams within the organisation start to evaluate other tools/approaches that fulfill a similar purpose to MBT, or if the costs associated with the MBT tests suddenly go up, or if model size gets to big, are, according to the interviewees, indicators that MBT is experiencing issues that could result in abandonment of the technique. Some statements were, “*Instead of fixing what is there [Technique/tool], you bring in something new and shiny and after a while you realize it doesn’t work either.*”, “*A warning sign is that the maintenance cost sneak off, especially in agile environments.*” As another example, one interview stated, “*Competence is important, if you are not competent you wont understand that the graphs can be too big. If a graph is too big, then we can’t understand how it will work. We can’t scale the graph too much either. One of the benefits of a model is that it is graphical and easy to understand. If you have one hundred boxes [vertexes] and a thousand arrows [edges] you won’t understand it or even care about it.*”. Measuring usage and controlling for such changes is therefore important for the longevity of the technique. These results show that there are multiple reasons and causes for abandonment of MBT. However, as stated by one of the interviewees in regards to the effects of abandonment, “*...as soon as you abandon something, it never becomes as bad as the doomsday prophets say. Everything works equally well anyways. There is huge amounts of survival instinct [within organizations].*”.

#### Summary for RQ3

Unlike the answers to the other research questions, less concrete results were identified, but two key aspects were still identified. First, a primary cause of abandonment is loss of knowledge, which is an *organizational challenge* that supports the need for adopting an organizational mindset for using the technique. As expressed by the interviewees, “*Be aware of champions leaving the organization if you havn’t reached the critical point for a large scale adoption.*”. Second, a couple of warning signs were identified which both relate to *management* of the MBT tests over time, e.g. mitigation of architectural or test degradation, and the cost of such management. For instance explained by one interviewee as, “*People get an eye opener for MBT from another part of the organization and see that the tests go red 30 percent of the time without changes. Can you make the tests more robust, in another/better way? You lift questions regarding test strategies and start to look over tools and their stability. Partly this resulted in the realization that some tests should not be done with MBT, but in other way. Cost-effectiveness/prioritization comes in [from a managerial perspective].*”. Combined, we note that root causes are on an organizational or team level, where loss of resources or knowledge influences the technique’s longevity.

### Summary of observed benefits and challenges

#### MBT benefits

The primary benefit of MBT models is test visualization, which improves communication between stakeholders and enables inconsistencies and defects to be captured earlier during development. MBT thereby fosters sharing of information, quality thinking and proactive defect identification, instead of reactive testing. Additionally, the technique is inclusive of new stakeholders since the models can convey test scenarios in an understandable way to non-technical stakeholders.

Another benefit of MBT is that it provides inherent architectural design—tests are specified top-down with a modular design as actions and events—fostering design-thinking and reuse. However, the interviewees stress that MBT test code must still be designed and well structured to be maintainable and reusable over time. Thus, stressing the need for development guidelines.

Yet another benefit of MBT is that tools have the capability to run models randomly. This enables MBT tests to emulate exploratory testing and thereby find new defects that were previously unknown. However, random model exploration for test generation/execution is associated with longer execution time than regression testing, and thereby higher cost.

#### MBT challenges

The following benefits and challenges were presented by several of the interviewees.

The main challenge with MBT is connected to the change in mindset that is required for MBT to be used effectively and efficiently in an organization. This requirement requires the entire organization to learn the technique and adapt their ways-of-working to incorporate MBT model creation, usage and maintenance. For older, more mature, organizations the adoption can be difficult since existing ways of working have inertia that prohibit change.

An associated challenge regards the definition of what a MBT test actually is, e.g. is a model a test or is a scenario a test? These questions may seem trivial, but in an industrial context MBT tests are compared to existing or traditional approaches through metrics or Key Performance Indicators (KPIs) reported up to management. A scenario that was presented in one of the interviews was that a MBT manager reported that one model had been completed and was running, which got backlash from higher management because it was compared to hundreds of tests reported by another team using another approach. While it may be obvious that comparing two different approaches should be avoided, it still happens. This presents additional challenges for adoption and use of the technique and requires comparable metrics to measure test performance, e.g. through metrics like coverage—a metric that most organisations use for testing. However, while code coverage could be measured, a question remains how higher level coverage metrics, e.g. feature coverage, should be measured.

Another challenge is that less impactful, smaller, software faults are more difficult/costly to manage with MBT than scripted, happy path, tests. The reason is because MBT tests are large and have some inherent complexity that provides good support for more complex scenarios. As such, MBT inherently supports functionality that is dichotomous to testing short, linear, happy path, sequences. This does not imply that MBT cannot test these sequences, only that other techniques may be better suited for it. Note that this statement does not contradict the previous statement that test sequences should be kept as short and simple as possible. Instead this observation infers that MBT sequences must not be too short according to the interviewees’ experiences. However, shorter scenarios may still serve a purpose since MBT can utilize them to create longer test sequences.

MBT test stability is also a challenge, where stability in this case refers to the tests’ ability to execute until completion. To achieve this in a good way, good engineering is required—models on suitable levels of abstraction and well structured MBT test code—but also stable development and testing environments. This challenge is not unique to MBT, but well-worth mentioning since the costs, expertise and organization of resources to combat this challenge are often not considered when adopting a new technique in practice.

## Best practices

In this section we present the best practices that were derived from the results of the study. The correctness of these practices were verified, through review, by industrial practitioners and interviewees that participated in the study. The participants thereby confirm that these are practices they have used or that they believe would be beneficial in practice. For the reader’s convenience, summaries of the 23 best practices can be found in Tables 2, 3 and 4.

**Table 2:** Summary of best practices for adoption of model-based testing derived from the conclusions and observations reported in Section 4

Phase	Connected to conclusion	#	Description
**Adoption**	Levels of abstraction	1	Evaluate employees based on their aptitude for abstraction before appointing them as MBT testers, set guidelines that clearly define suitable levels of abstraction and train personnel in using said guidelines.
		2	Appoint domain experts to model system behavior and have all stakeholders review the models.
	Mindset	3	Consider what changes that are required to the organizational culture for MBT to be efficient and effective.
		4	Consider what organizational changes that are required to make it easy for domain experts and technical personnel to collaborate with MBT.
		5	Ensure that the entire organization understands, and are knowledgable, about MBT, its benefits and drawbacks.
	Guidelines	6	Develop MBT guidelines for how to create models, write/maintain test code, version control MBT artefacts and the test environment.
	Test purpose	7	Evaluate the testing needs of the organization and ensure that MBT fulfils these needs.
		8	Ensure that there is need for random testing and/or combinatorial, scenario-based, tests on a higher level of system abstraction that makes MBT a suitable candidate for adoption.
	Re-execution	9	Evaluate the suggested MBT tool’s capabilities to automatically re-run generated test sequences. Alternatively, add such capabilities by constructing tools that can run MBT test logs or ensure that the MBT framework outputs executable log files. As a final option, ensure that outputted log files support easy, manual, re-execution of test scenarios.

**Table 3:** Summary of best practices for usage of model-based testing, derived from the conclusions and observations reported in Section 4

Phase	Connected to conclusion	#	Description
**Usage**	“Good” models	10	Adopt, if required, scenario-based requirements to better support modelling.
		11	Ensure that models are developed fit-for-purpose and that they are readable by all MBT stakeholders.
	Verifying MBT tests	12	Adopt model and test code reviews as standard practice for verification of the MBT tests’ correctness, design and quality.
	Maintenance	13	Create traceability matrixes that connect MBT tests to requirements and proactively maintain tests when requirements change.
		14	Update tests, models and code frequently to avoid quaity degradation, especially when tests fail.
		15	Run tests frequently to verify conformance between requirements, source code and tests.
	Test logic	16	Separate areas of concern for the tests to ensure that only necessary test logic is placed in the model. Otherwise such logic shall be placed in the test code to improve understandability of the tests.
	Return on Investment	17	Integrate MBT tests into the continuous integration pipeline/environment and automatically run the tests frequently (two to three times every day is suggested, but at least every night)
		18	Establish clear practices when the MBT tests are used for regression testing and when they are used for random/exploratory testing.
	Functional vs Non- functional tests	19	Consider how MBT can support testing of non-functional requirements (quality attributes) of the system under test, for instance performance tests.

**Table 4:** Summary of best practices for abandonment of model-based testing derived from the conclusions and observations reported in Section 4

Phase	Connected to conclusion	#	Description
**Abandonment**	Causes of abandonment	20	Ensure that the teams or individuals that are championing the MBT tests are given enough resources and mandate to manage the tests to avoid burning them out or otherwise loosing their competences.
		21	Ensure that the MBT tests, tools and practices are well documented and that the personnel is trained in the technique to mitigate the risk of knowledge degradation or loss.
	Warning signs	22	Be observant of projects that aim to adopt new/other test techniques or tools or make sure that these new approaches are fit-for-purpose as successors to MBT.
		23	Continuously measure the costs for maintenance of MBT models and test code and place additional resources on improvement efforts if costs suddenly increase.

### Adoption

Adoption, in this context, concerns the time-period from when MBT is identified as a candidate technique to fulfil the company’s needs until the time it is integrated in daily use at the company. The identified practices concern organizational-, cultural- and process-oriented aspects but also more tangible concerns for MBT use in day-to-day work. These practices thereby represent a checklist of items to consider as decision support *prior* and *during* adoption of MBT. The individual best-practices are summarized in Table 2 and described in the following sections.

#### Levels of abstraction

MBT models visualize the tests, making them understandable by all stakeholders. However, a prerequisite for unified understanding is that the models are developed on a suitable level of abstraction. This prerequisite requires the modeller to concretize/abstract information to the set level of abstraction, a skill which people do not inherently have. If possible, it is therefore of value to evaluate the aptitude for abstraction in the personnel working with MBT, for instance through a work test. Complementary guidelines, examples, training and management shall also be provided to ensure that models are created on (1) a suitable level of abstraction for the purpose of the test and (2) consistently kept on the right level. For 1, the suitability of a certain level of abstraction depends on the type of system and the type of test that is needed, for instance API tests require more detailed models than GUI tests. For 2, reviews and/or monitoring of the produced models are required. Especially in a context where models of models are used, where each subsequent model provides more details into a specific aspect of the SUT. Such design still requires each layer to be defined on a consistent and suitable level of abstraction. Consistency is important for the readability, understandability and tracability to both requirements and test code.

Modelling enables domain experts, with lacking technical knowledge, to read, write and understand the tests. Domain experts generally have the best understanding of the SUT’s intended behavior and are thereby the most suitable to develop the models. However, as technical design decisions, e.g. choice of third party components, can prohibit implementation of tests of certain system behaviors, it is important that all stakeholders are involved early in the adoption process and that models are reviewed by all stakeholders, including technical experts. Observe that *all stakeholders* does not imply all roles, but rather roles affected by decisions taken during the adoption and use of MBT. For instance, SUT architects can review model abstraction but should still, although not be responsible for, review MBT test code, which is better reviewed by developers or testers. In a best case, as mentioned by the interviewees, MBT is driven by the entire team, which in an agile context is cross-functional. Thus, ensuring that people with all necessary competences are aware of what, and how, MBT is used to test the SUT. These reviews serve to verify correctness and feasibility of the models but also serve as knowledge carriers to communicate technical requirements for the tests. Similar to traditional software development, technical requirements should specify what to develop, not how.

In summary, during adoption, it is crucial to involve all stakeholders in the adoption process to ensure that models are developed in a correct way and on a suitable level of abstraction. Roles mentioned by the interviewees include requirements engineers, test developers, testers, and quality assurance managers. However, the interviewees also mention that MBT is a team-based activity and that the team needs to be responsible. Key questions to ask at this stage are:

1. Are the models understandable by all stakeholders? Do they convey necessary information?
2. Is a single level model suitable or should a model of models architecture be employed?
3. Will the models be extendable and maintainable over time?
4. Do the models promote sufficient separation of concerns between test scenario logic and testing logic?

#### Mindset

To get the most out of MBT, the technique requires domain experts to collaborate closely with technical experts and other stakeholders. This way of working helps find defects and inconsistencies in the software specification early and promotes a quality-focused mindset throughout the organization. Transitioning an organization to this mindset can however be a challenge and takes time. To simplify the transition, information about, and rationales for, changes must be communicated throughout the organization.

In plan-driven organizations, or organizations with centralized requirements engineering, development, and testing teams, larger organizational changes may be required. Such changes include appointment of new roles that operate as interfaces between existing teams or new roles within cross-functional teams. In agile, commonly decentralized, organisations, similar changes may be required but should follow already established communication channels. Regardless, these changes are associated with considerable cost and should therefore be evaluated prior to adoption.

Additionally, collaboration between domain experts and technical personnel needs to be established. This may require the introduction of a common nomenclature to discuss MBT and to train the organization in how to use the technique, how it is implemented, and what its benefits and drawbacks are. This transformation is perceived necessary to establish the organizational mindset but also helps retain knowledge about the technique and its implementation in the organization over time.

However, a consequence of this guideline is that MBT success is reliant on adopting a MBT mindset, which may require significant organizational change on many levels of abstraction. For instance, but not limited to, addition of new, or changes to existing, roles, changes to communication among stakeholders (also vertically in the organization), new test development practices and management of the model artefacts. Hence, both the organisation (e.g. new or changed roles) as well as the process (e.g. ways-of-working and collaboration) may be affected. Such changes are perceived more difficult in larger organizations. This phenomenon can be expressed as organizational inertia, meaning that drastic changes take longer time to take effect. The adopting organisation shall be aware of this inertia and be prepared that the transformation may take time.

#### Guidelines

Similar to software development, it is important to define guidelines for MBT and train stakeholders to follow these guidelines. Furthermore, to ensure that the guidelines are followed, they should periodically, and systematically, be conformance tested within the organization.

These guidelines should include best programming practices, such as coding standards, naming conventions, version control, frequent maintenance practices and more. Additionally, key stakeholders and contact surfaces shall be documented to speed up communication.

The guidelines’ purpose is to maximize the effectiveness, efficiency and longevity of the MBT models and the MBT test code. This can be achieved with guidelines that promote consistency, coherency, readability, modularization, maintainability, extendability and testability of both the models and the code. As input for MBT guidelines, it is recommended to use the company’s existing guidelines for source code development. If existing guidelines are not available, general best practice guidelines shall be used.

#### Test purpose

MBT can fill various test purposes, including both regression and random exploratory testing. As such, MBT is a multi-purpose tool but this purpose is only achieved by the technique’s additional prerequisites and artefacts, e.g. models, which are associated with overhead costs. It is therefore important, from a cost and usability perspective, to prior to adoption consider what the testing needs of the organization are and for what purpose(s) MBT shall be used. In particular, the need for random, combinatorial, scenario-based tests should be evaluated and what additional value(s) these capabilities provide given the additional overhead costs of the technique. In particular, the combinatorial complexity of required test sequences shall be considered to estimate what value MBT can provide in terms of random/fuzz testing. If these mentioned MBT capabilities are not needed in an organisation, alternative test automation approaches—e.g. scripted testing or BDD, which are associated with less overhead—may be more suitable.

#### Re-execution

A primary strength of MBT is its ability to traverse the test model in random order, utilizing defined test sequences to emulate longer testing sessions and, if implemented, utilize random input data. This enables identification of erroneous corner cases and provides a good basis for non-functional testing, e.g. performance testing to expose memory leaks.

However, an observed drawback with longer test scenarios concerns how to replicate them when they find a failure, i.e. automated replication is not always supported by the tools and manual replication can be time consuming. To mitigate this challenge, it is important to evaluate if the proposed MBT tool has the ability to rerun random traversal of test scenarios or if such functionality can easily be added. Hence, prior to decision making of what tool to use, the following questions should be considered:

1. Does the suggested MBT tool support automated re-execution of random traversal of test sequences?
2. If no, can the tool export automatically executable logs or can the tool be extended with functionality to support automated re-execution?
3. If no, does the tool provide support for easy manual re-execution?

#### Usage

Usage of MBT, in this context, concerns the time-period after the technique has been evaluated, found suitable to fulfill the company’s needs, and integrated it into daily work. The proposed practices encompass design, management and verification of MBT tests for daily use. These practices, summarized in Table 3, are described in the following section.

**“Good” models:** The inputs used to design MBT models vary between companies, contexts and domains—requirements such as use cases, natural language requirements or other forms of requirements are used. However, since MBT tests are scenario-driven, it is recommended that scenario-based requirements, e.g. use cases, are used or otherwise adopted. Such requirements can more easily describe system states and state transitions, making modelling easier.

During MBT adoption, it is important that the model(s)’ level of abstraction and fit for purpose is considered and decided upon. However, during usage, the models’, and tests’, level of abstraction shall be fine-tuned and continuously improved to foster understandability, readability and maintainability. The models shall be seen as knowledge carriers between domain experts and technical experts of the SUT’s intended behavior. As such, the modelled behavior must frequently be evaluated and aligned with the SUT’s functionality and the requirements to ensure correctness of the tests. Note that this maintenance may not be required to remove deviations between the model and requirements but rather to optimize test flows, e.g. to improve coverage or to speed up test execution. Some key characteristics of a good model thereby include:

- Understandability - Easy to cognitively understand,
- Readability - Easy to read/interpret by all stakeholders,
- Fit-for-purpose - Defined on a suitable level of abstraction and properly defines system behavior,
- Maintainability - Easy to maintain and re-align with requirements,
- Extendability - Easy to extend with new test functionality,
- (Low) Complexity - Streamlined without clutter or unnecessary information/data/scenarios.
- Scaleability - The models shall be easy to extend, partition, etc. to pertain its usefulness as the system grows.

##### Verifying MBT tests

It is recommended that quality assurance of MBT models and test code are conducted with the same practices as source code. Examples of automated approaches (e.g. static code analysis and mutation testing) were observed in the study, but, due to their exotic nature and cost, they are not stated as a general recommendation. Test automation, e.g. unit or integration testing of third party components, contained in the test code architecture is however generally recommended.

Reviews is otherwise the premiered practice for quality assurance of MBT tests. The approach is common practice for source code development and has been shown to effectively finds faults and vulnerabilities in software engineering artefacts (Munaiah et al. 2017). Reviews can aid to ensure correctness, design and quality of both MBT models and MBT test code. These reviews should, if possible, be done with independence, i.e. by another individual than the creator(s). Similar to model development, model review is best served by domain experts, while review of test code is best served by technical experts. Specific questions to consider during review include, but are not limited to:

1. Is the model/test code easy to understand?
2. Is the model designed on a suitable level of abstraction?
3. Is the models’ size/complexity suitable for the test purpose?
4. Is the test code easy to read, is it annotated, does it adhere to coding conventions, etc.?
5. Is the model/test code extendable/maintainable/reusable/etc.?
6. Is the model supportive of both random exploratory testing and regression testing?

##### Maintenance

The majority of automated tests in industrial practice are used for regression testing. These tests aim to evaluate that a system, after change, still complies with requirements that were not changed. Similar to automated tests in general, MBT tests require continuous and frequent maintenance. This maintenance can be either proactive or reactive. Reactive maintenance, i.e. maintenance triggered by failing tests, is more common in practice but proactive maintenance, i.e. maintenance triggered by changes to the requirements, is proposed as a more effective, albeit less efficient, approach. As such, although proactive maintenance is recommended, reactive maintenance is considered good enough given that it is performed frequently.

To support proactive maintenance, a traceability matrix shall be used to establish traceability between the requirements, the MBT model and the test code. This matrix shall be frequently maintained and used when requirements, or code, change, to investigate if the model or test code needs to be changed as well.

Maintenance shall also always be carried out when tests fail to mitigate further quality degradation. The rationale for this practice is that repairing a single error in the model, or code, requires less effort than several. Hence, the longer between maintenance sessions, the larger the chance of faults masking each other, requiring further root cause analysis and thus greater effort to correct. Consequently, tests must be run frequently, especially if reactive maintenance is used.

##### Test logic

MBT test logic is separated between the test model and the MBT test code. Although the test code usually reflects the logic of the test model, in most tools MBT test code is a complement to the model, acting as the model’s driver. Thus, for the sake of the following discussion, we consider the two as two separate, but connected, entities.

A strong recommendation is to ensure that the two entities have different areas of concern. Whilst test logic that governs the test flows shall only be placed in the test model, all other logic, e.g. usage of libraries, special methods or assertions, shall be placed in the test code. This separation of concern ensures that all information required to understand the test sequences and/or system behavior is available in the model. Thus, making the model stand-alone readable, understandable, by non-technical stakeholders. In contrast, all other logic required to perform/drive the tests shall be placed in the MBT test code.

In the interviews it was argued by a few practitioners that all logic (including test specific logic) can be placed in the model. A counter argument, stated by other interviewees, is that such models quickly get cluttered. A cluttered model does not provide the same overview as a simple model, supporting the argument for a clear separation of concerns between model and test code. No guideline for the ratio between how functionality shall be divided between model and test code was identified in the interviews. As such, the ratio of separation, and allocated resources to develop each part, shall be determined by contextual factors.

##### Return on Investment

Return on investment (ROI) concerns the value provided, given the costs, of adopting and using MBT compared to other testing techniques. To achieve positive ROI, it is suggested that MBT tests are run frequently—preferably two to three times every day but at least once, e.g. nightly. The rationale for this suggestion is to get frequent feedback about the MBT tests’, and the SUT’s, quality. This is also proposed by guideline 14 in Table 3.

Note that this recommendation does not imply nightly random traversal of the model(s), but at least regression tests. The assumption made in this recommendation is that regression tests operate under stricter coverage criteria, e.g. cover each test sequence only once. Thus reducing overall execution time. For instance, vertex coverage is more time consuming than edge coverage and deciding a coverage threshold less than 100 percent coverage, per definition results in faster execution.

Random exploration shall still be run as frequently as possible, but this frequency is instead measured in days or even weeks depending on context. A recommendation is still to schedule the random testing and make sure that the organisation is aware of when/how these tests are run.

##### Functional vs Non-functional tests

MBT is mostly used for functional testing in practice. However, non-functional testing, in particular performance testing, can be achieved with the technique as well.

The interviewees had little experience with using MBT for non-functional tests, so detailed discussion is out of scope for this paper. However, from a higher level of abstraction, it is perceived that the following questions shall be considered when evaluating MBT’s use for non-functional tests:

1. What non-functional testing needs does the company have?
2. How can MBT cover the company’s non-functional testing needs?
3. What are the resource (e.g. time) constraints for the development, usage and maintenance of non-functional tests?
4. Who shall be responsible for the tests?
5. How, and how frequently, shall the tests be run to give feedback of suitable granularity?
6. Can the tests provide autonomous feedback or shall they be used together with other automated or manual practices?

### Abandonment

Abandonment, in this context, concerns when MBT tests have be used for a period of time but started to encounter challenges that point towards its abandonment. This implies removing, or replacing, the technique due to cost or quality concerns. The proposed practices cover both causes for abandonment and warning signs that abandonment could be a possible/suitable outcome. These practices are summarized in Table 4 and further described below.

#### Causes of abandonment

One of the most prevalent causes of abandonment of MBT is loss of knowledge and/or competence. In particular, when champions of the technique are lost, the technique is often abandoned soon after. This observation is not unique to MBT but rather a general concern for the adoption of any new technique, tool or practice in industry. Regardless, to safeguard the investment of adoption MBT, it is important to ensure that driving members of the organization are given enough resources and mandate to continue to use the technique.

Additionally, training, to spread the knowledge of how the technique is used and implemented is required to mitigate loss of knowledge. It is also good practice to document the MBT tests to mitigate the risk of knowledge degradation over time. This practice also helps with unavoidable turnover of personnel. However, since each of these activities are associated with overhead costs, they need to be budgeted in a suitable manner.

#### Warning signs

One warning sign that MBT is failing is that teams working with MBT start to evaluate other test approaches. Although this may be indicative of the teams exploring complementary approaches to MBT, it can also indicate that they are searching for a replacement. Regardless, if observed, the situation should be investigated to identify the root cause. The result of the investigation shall be a decision point where resources are either spent resolving the issues to salvage MBT or to facilitate expedient adoption of a replacement for MBT.

Another warning sign that can be measured quantitatively is the maintenance costs of models and test code. If this cost drastically rises, this is indicative of issues with the quality of the MBT implementation. For instance, the test code architecture may have not been sufficiently designed in the early stages of development, causing technical debt, which is in later stages causing excessive maintenance efforts to be required. Alternatively, the test models may have been designed on an unsuitable level of abstraction. Yet another cause can be that the models have simply grown to large for the modelling team to keep up with changing requirements. Most of these challenges can be mitigated by additional resources, but to be sustainable long-term, the MBT practices need to be scaled appropriately as the SUT scales.

## Discussion

The take home message of this work is that MBT has both unique and, to other techniques, common preconditions and conditions that affect the value and benefits of the technique’s use for quality assurance in industrial practice. The guidelines proposed in this work provide guidance to highlight these conditions and give suggestions for practices or artefacts to include in the MBT testing process.

In terms of benefits, firstly, the technique promotes a collaborative way-of-working that is associated with early fault detection. Second, the technique is multi-purpose, meaning that it can be used for both random exploratory testing and regression testing. Third, the test models provide inherent test architecture, high flexibility and reuse. Fourth, the technique can be adapted to work on most levels of system abstraction given the right test drivers.

However, the technique also has challenges, such as the need to change organizational mindset to foster more communication and collaboration. This challenge is significant due to the costs associated with the transition in mindset but also because this change is connected to the primary benefit of the technique.

Second, graphical modelling is different from most common coding practices and therefore alien and more difficult for some stakeholders to adopt. In a worst case, this can cause friction in the workplace.

Third, random test exploration is associated with longer test execution times. This challenge raises a question regarding the technique’s suitability in a modern, continuous integration, environment where rapid feedback is essential for agile development, i.e. do the time constraints of modern development environments accommodate MBT? An additional challenge with random exploration is replication—effective and efficient re-execution of randomly generated test scenarios that identify faults. Not all MBT tools/frameworks support automated re-execution, leaving it to developers to either add or create this functionality themselves or manually replicate the test scenarios. Hence, costly solutions that may be seen as deal-breakers for many organisations interested in the technique.

Comparing these benefits and drawbacks against each other, the benefits of higher quality and automation look to come out on top. Still, when surveying the adoption of the technique in industry, we note sparse usage compared to the interest the technique has received from academia. The interviews gave some insights to explain this phenomenon. First, the drawbacks of cost, organisational change and mindset, are significant challenges that are showstoppers in many organisations. Furthermore, knowledge about MBT is lacking in industry and there are many misconceptions about the technique, its usage and value. Instead, the technique is often viewed as exotica, excluded in favor of techniques with similar testing purpose, discussed later in the paper. Hence, although MBT is well known in testing research, according to the interviewees, this research knowledge has not been properly disseminated into industry.

Another challenge that limits industrial adoption is a need to “sell” the approach to the organization. Hence, practitioners need to lobby for the technique to get resources to evaluate and/or adopt it. In particular, connected to the aforementioned change in mindset, “getting everyone on board” with the technique was presented by several interviewees as a challenge. Whilst this challenge is not strictly a research problem, it is connected to the spread of knowledge of the technique in industry, such as guidelines for MBT best practice.

The interviewees also brought up several competing techniques that can substitute MBT with graphical models, e.g. exploratory testing, scripted testing and more. However, the most prevalent, and surprising was Behavioral driven development (BDD) (Irshad et al. 2021) with scripts written in Gherkin (North 2010) syntax. BDD is also considered a model-driven approach, but, instead of graphical models, it relies on textual models to describe feature requirements. The textual approach makes BDD more common to the developers’ normal ways-of-working and this commonality is explained as one factor that influences its industrial success over MBT with graphical models.

Furthermore, although MBT has unique characteristics, it has many commonalities to other test automation approaches. These commonalities relate not only to the technique’s capabilities, but also its hindrances and challenges. From a capability perspective, MBT is, despite its additional artefacts and steps, equally suited for test automation as other techniques, e.g. scripted testing. Similarly, aspects like “getting people on board”, required knowledge and training, high maintenance costs and warning signs for abandonment, can all be observed for other techniques. For instance, sudden rise in maintenance costs is considered a warning signal for MBT heading towards abandonment. Similar results have been reported for other test automation techniques in literature—At Spotify, Visual GUI Testing was abandoned due to increased maintenance costs (Alégroth and Feldt 2017).

### Implications

The implications of this work are divided into contributions to research and contributions to industry.

#### For research

The study provides empirical evidence from experts around the world on the benefits, drawbacks and challenges with MBT. Some of the challenges, for instance test re-execution, are technical challenges were research could contribute with solutions. For instance, ways of capturing random test execution in a better way, methods to achieve test scenario minimization, and perhaps novel analysis methods. For other challenges, like changing organizational mindset, research could contribute with effective and efficient models and guidelines how to adopt and use MBT. This paper makes an attempt at formulating such guidelines, but further empirical evaluation and refinement is required. Future work will be discussed further in Section 6.3.

Another important outcome of this work is that MBT has not been communicated well enough to industrial practice. This was pointed out in the interviews, i.e. that many practitioners don’t even know that MBT exists, even less how to use it. Here, academia, given the vast body of research on the technique, could step up to help industry more clearly see the value of the approach and thereby achieve better penetration in practice. For instance, more experience reports and success stories from different domains could be of great benefit. Other efforts include more industrial research in co-production, higher presence at industrial conferences and more practitioner-oriented publications.

#### For industry

The proposed guidelines provide decision support and guidance for companies seeking to adopt MBT but also for retrospective analysis on past adoption attempts. For adoption, the guidelines provide insights into important aspects that should be evaluated and decided upon to prepare the organization for long-term use of the technique. For companies that have previously abandoned the technique, the guidelines can give insights into why the technique failed, and if MBT was reintroduced, how to do so differently.

Finally, for companies already using the technique, the guidelines present aspects to continue to improve upon in terms of usage, but also warning signs to consider to prevent the technique from being abandoned. Whilst many of the guidelines should be obvious to practitioners that have worked with MBT, others, for instance that not everyone has an aptitude for model abstraction, may give valuable insights into issues that their organization are struggling with. Hence, whilst this work includes results on technical aspects connected to MBT, e.g. to consider how to partition test logic between models and code, it provides also a contribution in presenting other aspects relevant for successful MBT adoption and use. These aspects include both organizational aspects, e.g. the need for more collaboration, but also process aspects, e.g. when/how to run the tests as part of continuous integration. Note that the guidelines try to avoid stating “how” to achieve improvement, rather, they focus on “what” aspects to consider.

### Threats to validity

There is, as with most empirical research, some threats to consider in regards to the results of this study. This section aims to bring attention to these threats and discuss their implications. For this discussion, the four concepts of internal validity, external validity, construct validity and reliability, inspired by the guidelines set by Runeson et al. (Runeson and Höst 2009) have been considered.

#### Sample (external and construct validity)

The interview sample for the study is small (N = 17) when compared to the sample-frame of potential, but unknown, number of MBT users. The sample makes up for this by consisting of experts with many years of experience with the technique from multiple companies, contexts and domains. The sample is also drafted from different parts of the world, implying that contextual factors, like geographic norms, can be excluded. The sample’s expressed experiences with the technique in practice are also mostly similar, supporting the validity of the synthesized results. However, although heterogeneous, there is a risk that guidelines may have been missed. We therefore do not claim these guidelines to be comprehensive and urge more research in this area, including research into non-technical factors associated with the technique.

#### Delimitation of the results (external validity)

Although the results are perceived generalizable to software intensive systems, due to the sample’s heterogeneous experiences, there is a lack of results from some known MBT domains such as the automotive domain. This is perceived to delimit the results’ generalizability. However, since MBT is primarily underrepresented in the software intensive software development market, which is the focus of the study, this delimitation is found acceptable. It is possible that the guidelines can be applied in contexts not represented by interviewees’ statements, but contextual differences cannot be excluded. For instance, embedded systems have technical dependencies on physical components, adding new requirements and stakeholders to the MBT environment, which may prohibit the described ways-of-working from being used. MBT’s use case must also be considered. As an example, in the automotive domain, MBT is used to model signal behavior in, for instance, electronic control units (ECUs). Hence, modelling on a lower system level of abstraction than discussed in this paper. This may invalidate some of the guidelines discussed in this work. For example, when modelling low-level technical behavior, is it still suitable for this modelling to be conducted by domain experts rather than technical experts?

#### Research procedure (internal validity)

The procedure of drafting, piloting, conducting, analyzing and verifying the interviews’ results were inspired by academic best practices (Runeson and Höst 2009; Azevedo et al. 2017; Baskarada 2014). These steps were performed by the research team, with varying experiences and knowledge of research, MBT and domain knowledge. Despite the heterogeneous skill set, it cannot be ruled out that the research team were flavoured by the study objective, leading to biases. However, since the results were verified by external experts, such biases are considered minor, if present.

The results are also evidence-based, i.e. derived from multiple sources (interviewees), and triangulated (Runeson and Höst 2009; Baskarada 2014). Additionally, the results answer the research questions but, as stated previously, these answers are delimited to development of software intensive systems and MBT with graphical models.

#### Replicability (reliability)

This case study can not be replicated exactly because the responses from interviewees may vary. We’ve therefore provided detailed descriptions of the research procedure and the decisions taken to reach the results and discussed the possible limitations of these. Interview questions have also been presented as well as the step by step analysis procedure. Hence, we expect the reader to be able to follow the design, judge its, and the conclusions, validity. Please note that although other guidelines could result from another sample of interviewees, we expect there to be significant overlap to our result. We base this claim on the characteristics of our sample and their suitability for the study.

### Future work

Several avenues of future work can be seen as a result of this work. First and foremost, there is a lack of experience reports about MBT in practice. Such reports are required to spread best practices and success stories to motivate, inspire and educate industrial practitioners about MBT. Further work is also required to spread awareness of the technique in non-academic forums, such as industrial testing conferences.

However, there is also an alternative aspect to consider; it is possible that MBT is not a suitable tool in most software-intensive domains when compared to other available test techniques. This hypothesis leads to an interesting question that should be explored comprehensively through empirical research; what are the benefits of MBT with graphical models compared to other techniques such as BDD? The answer to this question could help explain why, for instance, BDD has seen increased popularity in industry compared to MBT. One hypothesis is that the lack of MBT adoption is related to MBT’s prerequisites, e.g. organizational mindset changes, but this conclusion should be explored further.

However, assuming that MBT has a place in industry, it is evident that ways-of-working with the approach and best practices are still required. This paper makes an initial attempt at presenting non-technical guidelines but it is not perceived to be comprehensive. For instance, it is unlikely that the results touch upon all organizational and human factors associated with MBT’s use in practice. In addition, few results were acquired regarding the technique’s use for testing non-functional requirements. Hence, more industrial studies are required to elicit well-tested and used practices such as those presented in this work. The goal of such research should be to build a more comprehensive model of the use case, challenges and ways-of-working with the technique to stimulate further industrial adoption.

## Conclusions

Model-based testing (MBT) has been extensively researched for several decades, resulting in technical advancements as well as practices and ways-of-working. However, despite the extensive body of knowledge, MBT is an underrepresented technique in practice. One reason has been identified as the lack of knowledge about the technique due to lack of empirical studies, e.g. success stories, and evidence-based guidelines that capture a broader perspective than the technical aspects of the technique.

For this paper we performed 17 in-depth interviews with MBT experts from around the world to elicit the experts’ knowledge, best practices and experiences with MBT. The interview resulted in 13 high-level conclusions that were transformed into 23 best practices for the adoption, use and abandonment of MBT in practice. The results cover both technical and non-technical factors and thereby provide a novel contribution in terms of evidence-based, broader encompassing, guidelines for MBT.

A synthesis of the perceived more influential conclusions of this work are summarized below:

1. MBT requires models to be designed on a suitable level of abstraction but many individuals lack an aptitude for abstraction.
2. MBT requires a change in organizational mindset with more collaboration among stakeholders to be successful. However, once established, this collaboration is also MBT’s main benefit that allows the technique to support early fault detection, even without test execution.
3. MBT is a multi-purpose tool, supporting both regression and exploratory testing, but if multiple purposes are not required by an organization, then other techniques may be more suitable.
4. MBT is subject to many of the same challenges as other test techniques when it comes to “getting everyone on-board”, warning signals that it is not working, and prerequisites for operation (resource and knowledge requirements).
5. MBT inputs and test design can vary but a general recommendation is to use scenario-based requirements as inputs and put all logic that governs the test sequences in the test model, whilst all other logic is placed in test code.
6. MBT test modelling and execution is not considered a challenge since it is well supported by existing MBT tools. However, re-execution of random traversal of models is considered a challenge.

Along with these results, we conclude that further research on the areas of adoption, usage and abandonment of the technique are required and outline four key areas for such research, i.e. more industrial experience reports, comprehensive analysis of the benefits/drawbacks of MBT compared to other automation techniques, further work into non-technical guidelines and more research into how to utilize MBT for testing non-functional aspects of a SUT. We hope this study will inspire both industrial practitioners and academics to continue to pursue knowledge about MBT and spread awareness of its capabilities in industrial practice.

## Appendix A: Interview Guide

In the following, the interview guide used during the study is presented. The interview questions are

### Before interview

- **Explanation of study’s purpose:** The purpose of the study is to develop guidelines for how MBT should be used in practice.
- **Statement of anonymity:** All answers will be anonymized and no answer will be traceable back to you.
- **Approval to record:** Do you agree to this interview being recorded? The recordings will be held confidential and will not be shared outside the research team. No information you provide will be given to any of your colleagues or managers before point (2) has been fulfilled.
- **Statement of possibility to review:** You will be given the possibility to review all materials before they are published/presented to the public.
- **Clarification of study scope:** MBT stands for Model-based testing and is in this interview defined as automated tests that are produced through graphical models developed in a modeling tool that are connected to executable, automated, tests consisting of code.
- **Requested data:** We primarily seek empirical experiences and knowledge from actual use of MBT in industrial practice. Please clarify if your response is based on your own perception or if your answer is theoretical.

### Background

1. How many years of industrial experience do you have in the software development industry?
2. How many years of industrial experience do you have of software testing (manual or automated)?
3. How many years of industrial experience do you have of MBT?
4. As an MBT user, what was/is your role (developer, tester, manager, etc)?

### Adoption

1. Which testing needs are covered by MBT that made the approach attractive for the projects you have participated in?
  - (follow-up) Did you seek to replace or complement existing tests with MBT?
2. Which changes to the work environment are required to make MBT work in daily work?
3. Which roles should take part in the adoption of MBT and what are their tasks?
4. Which are the challenges with adopting MBT into daily work?
  - (Follow-up) Was there support throughout the organization for MBT as a technique/concept?

### Use

1. Which roles shall be responsible for the development, quality assurance and management of model-based tests and what are the tasks of these roles?
2. Which aspects do you consider when choosing MBT as the most suitable technique to create a test case? (Given that you have MBT in your tool box).
3. Which guidelines shall be used to succeed with model-based testing? (*Each of the following sub-questions shall be asked*).
  - How shall input for model-based tests look like and how shall it be used?
  - How shall test-functionality be split between model and code?
  - How much time shall be spent on creating a model compared to time spent on making code?
  - How shall the development of models and code be split between different roles?
  - How shall model-based tests be maintained to ensure their longevity?
  - How often shall model-based tests be executed to give return on investment?
  - How shall models/code be quality assured?
  - How do you collaborate and reuse code/models in an effective/efficient way?
  - How do you recreate test flows for troubleshooting when MBT reports a fault?
  - How do you use the outcome from MBT tests in the best way (reports, results)?
4. What type of functional- and quality- (non-functional) tests shall MBT be used for?

### Abandonment

1. Which causes lead to MBT abandonment and what are the warning signs that indicate this outcome?
  - **Note for follow-up questions:** Consider loss of competence, failure to introduce MBT in CI, contextual problems.
2. Which effects does abandonment of MBT have on daily work?
3. Which techniques/tools are most suitable to replace MBT after abandonment?
4. Which changes would you do to better succeed with the re-introduction of MBT?

### Other

1. Do you have any additional comments about MBT that you think we have not covered in the interview?

## Footnotes

1 Abandonment considers warning signs that abandonment may be an outcome and practices to replace MBT after abandonment.

2 Special care was taken when analyzing the first pilot interview to avoid bias.
